# Towards a systematization of brain oscillatory activity in actions

**DOI:** 10.1038/s42003-023-04531-9

**Published:** 2023-02-02

**Authors:** Christian Beste, Alexander Münchau, Christian Frings

**Affiliations:** 1 Cognitive Neurophysiology, Department of Child and Adolescent Psychiatry, Faculty of Medicine, TU Dresden, Dresden, Germany; 2 Institute of Systems Motor Science, University of Lübeck, Lübeck, Germany; 3 Cognitive Psychology, University of Trier, Trier, Germany

**Keywords:** Cognitive control, Human behaviour

## Abstract

Information processing in the brain is governed by oscillatory activity. Activity oscillations in specific frequency bands (theta, alpha, beta and gamma) have been associated with various cognitive functions. A drawback of this is that the plethora of findings led to considerable uncertainty as to the functional relevance of activity in different frequency bands and their interrelation. Here, we use a novel cognitive-science theoretical framework to better understand and conceptually harmonize neurophysiological research on human action control. We outline how this validated starting point can systematize and probably reframe the functional relevance of oscillatory activity relevant for action control and beyond.

## Abstract (web summary)

Beste and colleagues propose a theoretical framework that reconsiders the functional relevance of oscillatory activity in the context of action control.

## Introduction

Information processing in the brain is governed by oscillatory activity. In the past decades, a wealth of empirical findings has clearly underlined the importance of synchronized oscillatory activity, for many cognitive functions including perception, attention, learning, memory, motor control and higher-level goal-directed behavior. However, the plethora of neurophysiological findings^1^ associated with cognitive functions has caused considerable uncertainty as to the functional meaning of activity in different frequency bands and also their inter-relation—even though first attempts to systematize this have been made^1^. Yet starting from a neurophysiological basis is problematic when it comes to interpretative confidence because neural processes are, due to their complexity, often only weakly conceptually constraining^2^. Explanatory frameworks tailored to specific paradigms/phenomena and research areas can contribute to considerable fragmentation in the interpretation of neurophysiological findings impeding scientific progress^3^ and rendering interpretations of the functional significance of oscillatory activity difficult. There is thus an urgent need to increase efforts in theory-building^4^ and to better link psychological concepts with neuroscience^5^ to provide more overarching and theoretically stringent interpretations of oscillatory brain activity underlying cognitive functions in general. While theory-building in general is not without problems because multiple ‘parallel’ theories can lead to a scientific impasse ultimately leading to more confusion than clarity, the way we suggest to pursue this is unlikely to lead to such problems. The reason is that the approach we take does not provide a novel theory at the same level or conceptual breadth of other theories in the field. Rather, it specifies working principles and broadens an existing, well-validated theoretical framework. We pursue this goal using an emergent cognitive science framework conceptually harmonizing research on human action control and related fields—the Binding and Retrieval in Action Control (BRAC) framework^6^ which stands in the tradition of the so-called ideomotor theory (see Box 1)^7–9^. We will outline how this validated cognitive science framework may be useful as a starting point to systematize functional interpretations of oscillatory brain activity in different frequency bands that have been found to be involved in action control.

Box 1 Glossary of conceptual terms and definitions Ideomotor theory: Continuously picking up contingencies between own activities and its impact on their environment (i.e., to integrate perceptual and motor processes) is the core of the so-called ideomotor theory. The central assumption is that for an action to be accomplished one first has to anticipate the perceptual effect the action will produce. Action-learning enables to understand the relation between movements and their sensory effects, which in the end leads to the ability to act on purpose.

Action control tasks: Experimental paradigms to tap a specific aspect (e.g., response inhibition, distractor-based retrieval, planning etc.) in action control. Most of these tasks are sequential in nature, that is, the procedural structure comprises a prime-display followed by a probe-display. Performance is typically measured at the probe. BRAC emphasizes that binding/integration takes place at the prime and that upon feature repetition at the probe retrieval reinstates the prime-event file. Typical and prominent examples of these tasks are task switching, flanker tasks, Negative Priming, S1R1-S2R2- tasks, motor planning tasks, and distractor-response binding tasks etc.

Theory of event coding (TEC): The basic idea is that perceived and produced events (stimuli and responses) are cognitively represented in so called event-files (i.e., episodic representations of events) and that these representations interact to generate all kinds of perceptions and actions. TEC is a very general framework that explains the modulation of action due to retrieval of previously established event-files. In addition, the planning and selection of actions due to anticipated action-effects trigger the motor programs that were formerly integrated with these effects in event-files.

### The BRAC framework

**Fig. 1:**
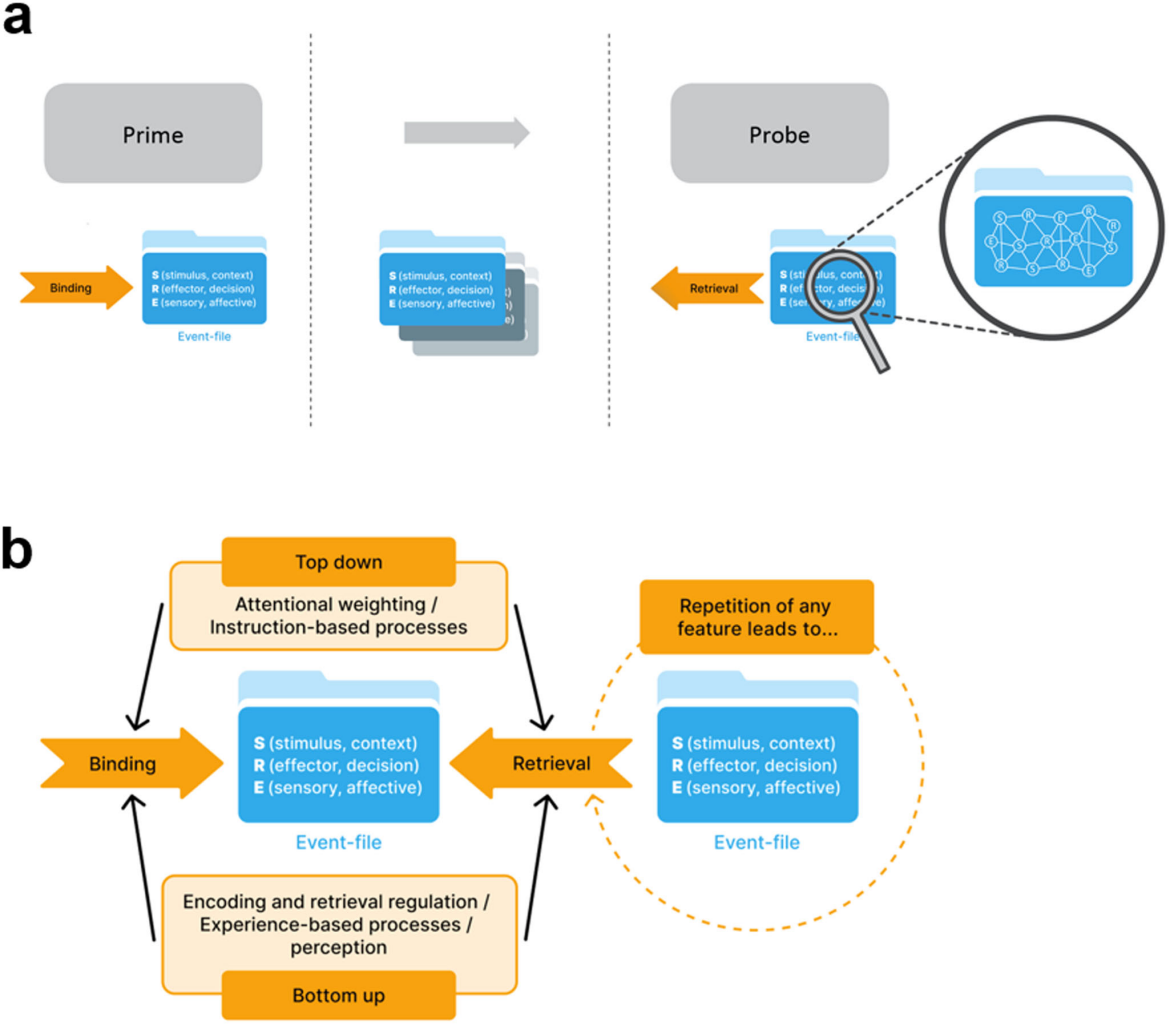
Principles of the BRAC-framework. **a** Characteristics of commonly used experimental tasks to examine action control. Most tasks reveal a sequential structure (a trial n-1 to trial n structure), i.e., two consecutive displays are presented – the prime display followed by the probe display. Behavioral performance as well as neurophysiological processes underlying action control are typically examined at the probe. Crucially, BRAC states that binding/integration takes place at the prime and that upon feature repetition at the probe retrieval reinstates the prime-event file. **b** Schematical illustration of the BRAC framework delineating how event-files are managed. The event-file is at the core of the framework, which assumes two central processes: (i) the binding of stimulus (S), response (R) and effect features (E) into the event-file. (ii) a retrieval process of a previously bound event-file whenever one of the S, R or E features is re-encountered. This reflects the retrieval of an episodic memory trace. Importantly, binding and retrieval processes work independently from each other and are both subject to top-down or bottom-up modulatory effects.

Action control research is concerned with the understanding of how humans plan and execute actions. Actions are one of the most important outcomes the cognitive system can accomplish. Through actions creatures shape, influence, and interact with their environment, including other creatures^10^. The BRAC framework^6^ was developed against the background that research in action control is fragmented into different paradigms/tasks (see Box 1) and paradigm-specific explanatory concepts, each covering a particular aspect of action. For instance, task switching investigates flexibility^11^, negative priming a facet of inhibitory control^12^, stimulus-response binding tasks binding of features^13^, and sequential modulations of Stroop or flanker effects cognitive control (i.e., the Gratton effect). Crucially, such typical experiments to examine action control are all *sequential* in nature, that is, they comprise a prime-probe structure (or trial n-1 to trial n structure). Given this sequential structure, i.e., the procedural equivalence of all these tasks, it is possible to integrate action research into a single comprehensive framework, the BRAC framework^6^ (see Fig. 1) and use this to also develop a theory-driven nomenclature of the functional relevance of brain oscillatory activity underlying action control.

According to BRAC, features of the stimulus configuration (stimulus, context, cue), the response (response goal, decision, effector), and the effect (sensory and affective) are integrated into an episodic memory entry that is labeled ‘event-file’ according to the *Theory of Event Coding* (TEC) (see Box 1)^14^. An event-file^15^ constitutes an internal representation of stimuli, responses, and effect features. The concept of an event-file follows the tradition of Kahneman’s and Treisman’s object files that consisted only of stimulus features. In an event file, all features describing a stimulus (S), the associated motor response (R) and the likely effect (E) are stored in a way that each stimulus feature (S) becomes associated (bound) with each feature defining the response (R), and its effect (E). Event-files can also comprise context (C) or distractor (D) features^16^. Event-files deacy quickly over time albeit translation into long-term memory is possible^17^. Upon repetition of any feature of a previously established event-file, the event-file containing this feature is retrieved facilitating the execution of the action encoded in this event-file.

Binding and retrieval are treated as separable processes in the BRAC framework. Put differently, BRAC conceptualizes action control in terms of dynamic event-file management (integration versus retrieval of event-files). As regards the above-mentioned action control tasks, event-file binding occurs during trial n-1/the prime and, in the case of feature repetitions, retrieval at trial n/ probe onset. The typical so-called ‘binding effects’ or ‘action effects’ that emerge in these tasks are, according to BRAC, always a composite of binding proper *and* retrieval. In addition, both top-down and bottom-up processes separately influence binding and retrieval. For instance, top-down control can modulate processes of binding and retrieval through attention weighting, or varying semantic representation (e.g., task rules, framing, mind sets, speed/accuracy tradeoffs, instruction-based effects). These control processes can exert influences on the binding process (e.g., features receiving more attention might be more likely to become integrated into event-files^15^) and/or the retrieval process (e.g., features that are ignored might be less effective retrieval cues^18,19^). The bottom-up modulation of binding and retrieval reflects effects that are stimulus-driven or experience-based, like influences of contingencies^20^, affective states^21^, and perceptual configurations^16^. Therefore, BRAC can integrate paradigm-specific findings and replace paradigm-specific conceptual approaches on action control strongly dominating the field.

Importantly, BRAC’s reach goes beyond the field of action control^6^. Many well-known effects in research areas related to action, namely attention, memory and learning, as well as motivation can also be separated into instances where the recent past (event-file binding) shapes current behavior (due to event-file retrieval). In other words, these areas can also be interpreted in terms of feature binding and retrieval parts, significantly increasing the reach of BRAC and its conceptual relevance in cognitive neuroscience. Take Pavlovian conditioning as an example. It is a well-known paradigm in the memory and learning research field seemingly unrelated to action control. In Pavlovian conditioning, incidentally pairing (or binding in BRAC terms) a formerly neutral (conditioned) stimulus with an unconditioned stimulus eliciting an unconditioned response endows this conditioned stimulus with a tendency to trigger (or retrieve in BRAC terms) the same response on a later occasion, even in the absence of the unconditioned stimulus.

The central aspect of BRAC is that paradigms (e.g., action control tasks) are only vehicles to study/try to capture psychological constructs and paradigms are, in the field of action control, highly structurally similar and thus examine highly similar cognitive processes. It is the focus on paradigms that have been thought to measure different aspects related to action control - which is unlikely to be case—that has led to a considerable fragmentation of research in action control^6^. Since BRAC can abstract from the specific paradigms through its focus on the structural similarity of different paradigms, BRAC has a high generalizability. Therefore, and instead of designing more new paradigms (action control tasks), it is important to provide more clarity of what is already existing and synthesize this.

In fact, many lines of research have already corroborated the conceptual validity of the BRAC framework, and hence its usefulness as a starting point to re-conceptualize and systematize the neurophysiology of action control and related processes. For instance, the main assumption of BRAC, that binding, and retrieval should be treated as separate processes each contributing independently to observed behavior, was confirmed. In particular, perceptual variables like salience or figure-ground segmentation have been found to exert their influence particular on binding^22–24^, while temporal variables exert their influences independently on binding and retrieval^25–31^. Predictability of stimulus and effect contingencies^32–36^ were used to manipulate binding versus retrieval separately. In addition, perceptual grouping has been successfully used to manipulate binding versus retrieval^37,38^. Furthermore, context manipulations that were applied separately to binding versus retrieval aspects again suggest that these processes should be treated as independent process^39–41^. Together, these findings suggest that binding and retrieval can be experimentally separated (by applying previously known modulators of action control separately to the prime display where binding happens or to the probe display where retrieval happens) and reflect different processes separately contributing to behavior. What is more is that recent EEG papers found correlations between brain oscillations and one of these particular processes. For example, the binding process has been related to BBA while retrieval processes seem to be related to TBA (see below). Against this background we suggest that the BRAC framework might be a novel approach to re-structure the literature on oscillations in action related fields and to connect cognitive science and neurosciences^5^.

### BRAC’s potential to systematize the neurophysiology of action control and related processes

A general asset of a good scientific theory is that it’s concepts inform the data collection process by determining what data is collected to best capture the theoretical content and help constrain the conclusions drawn from empirical research^42^. Regarding this, the asset of BRAC is its potential to summarize and integrate a vast amount of paradigm-specific research findings and theorizing in the field of action control and to integrate many well-known effects in areas of research *related* to action. Crucially, this renders BRAC a versatile means to organize research in the neurophysiological underpinnings of action control due to its reliance on the event-file concept.

BRAC effectively extends the Theory of Event Coding (TEC) (see Box 1) by placing the ‘event-file’ construct in the center of the framework and explaining action control by dynamic event-file management in terms of event-file binding and retrieval. The event-file reflects as a network of distributed representations^43^ likely constituted by one or a combination of the three following mechanisms: “Integration by Convergence”, “Integration by Correlation” or “Integration by Indexing”^15^. The first mechanism relies on neural units that are selective for the presence of particular sensory or motor feature combinations. The second mechanism relies on synchronizing the firing patterns of neural units representing features of the same event^44^. Synchronicity could increase the impact of the synchronized unit on other processes (e.g., perceptual or response processes). The third mechanism may work by enhancing the firing rates of sensory related neural units^45^, which support the creation of adaptive links between sensory- and action-related feature codes^46,47^. This role has been associated with neural units in the prefrontal cortex^48,49^ and the parietal cortex^50,51^. A common theme of these possible mechanisms leading to the emergence of an event-file is the relevance of oscillatory activity, and its synchronization, as captured by electrophysiology^52^. Since event-file dynamics are central for BRAC, and because BRAC has a *process-oriented* nature, BRAC can be useful to constrain and likewise refine interpretations on the functional relevance of oscillatory activity in action control. While there has been some debate in the past about whether oscillatory activity only reflect an epiphenomenon, it is generally accepted that neural oscillations represent one of the fundamental mechanisms of cerebral functioning^53–55^, which also becomes evident from a biophysical point of view. According to the “temporal binding hypothesis”^56–58^, information processing between distant neural assemblies depends on the strength of a coherent organization of activity through synchronous neural oscillations^59^. Similar aspects are also stated by the ‘binding-by-synchronization (BBS)’ hypothesis and the ‘communication-through-coherence (CTC)’ hypothesis^60^, according to which effective communication and integration of information is implemented by the pattern of coherence among neuronal assemblies, i.e., the pattern of phase-locking among oscillations in the communicating neuronal groups^60^. Communication between two neuronal groups depends on neuronal coherence between them and is prevented by its absence^60^. Synchronization of neural activity is essential for the dynamic coordination of distributed neural activity in local and extended networks underlying various cognitive processes^59,61,62^. Information processing between distant neural assemblies strongly depends on the strength of a coherent organization of activity through synchronous neural oscillations^54,59^. Higher frequencies (such as gamma band activity >30 Hz) may serve the generic cortical computations underlying local encoding of information^63^, while long-range interactions tend to involve a larger spectrum of frequency bands comprising theta (4–6/7 Hz), alpha (8–12/13 Hz), and beta (13–25 Hz) frequencies^64^. Particularly low-frequency, high-amplitude oscillations are suitable to integrate information across spatial distances^59^, because lower frequencies put fewer constraints on the precision of timing since the phases of increased and reduced excitability are longer^65,66^. Crucially, several lines of evidence suggest that signatures of oscillatory activity are highly preserved across species and brain sizes^67^. Considering that BRAC roots in ideomotor theory^6^ and thus in principles being evident across (mammalian) species^7^, BRAC principles can, therefore, provide a framework for research on animals and humans alike. In the following we re-organize the literature with a focus on the modulation of low-frequency bands (theta, alpha, and beta) during action control from a BRAC perspective.

### Theta and gamma band activity in the light of BRAC

Theta band activity (TBA) has been subject to intense research. Evidence for an involvement of TBA in action control has been corroborated by various studies using different paradigms. In recent years, a number of studies have corroborated a role of TBA activity properties in event-file processing, either by examining TBA directly using time-frequency EEG data^68,69^, or indirectly through EEG responses in the time domain known to depend on TBA^69–75^. In line with biophysical properties of TBA^59^, and its original conceptualization in TEC^15,43^, event-file dynamics reflect processing in a distributed cortical and subcortical network encompassing inferior and superior parietal areas, supplementary motor areas, the dorsolateral prefrontal cortex, and the hippocampus^69–76^. Theta network dynamics during event-file processing are modulated when demands on action control processes increase. Along the same lines, event-file reconfigurations modulate theta and decrease alpha-band activity^68,69^. Vice versa, when there was no need to reconfigure perception-action bindings, TBA was low and alpha-band activity (ABA) was high. This shows how perception-action integration is affected by (preceding) transient neurophysiological connectivity states.

Influencial work has suggested that especially the role of medial frontal cortex TBA in action control may emerge from biophysical properties TBA^77^, i.e., that particularly low-frequency, high-amplitude oscillations are suitable to integrate information across spatial distances^59^. Action control critically depends on the proper integration of perceptual and motor aspects, which is also reflected by the event-file concept^14,15^ as the core of BRAC. At present, this BRAC-related potential conceptual role of TBA has not been taken into account sufficiently in studies on action control. This notwithstanding, it has become clear that TBA underlies various instances of action control processes^77,78^ that BRAC seeks to explain using a small testable set of binding and retrieval processes^6^. It has been suggested that TBA during action control processes serves as a “surprise signal” necessary to adapt actions^77^. Such theta-related surprise signals would be used to alter learning processes by shifting behavioral strategies, and to increase cognitive control^77^. However, the computation of a surprise signal necessarily depends on a comparison process of incoming information and stored information building the basis of expectations. Whereas TBA has in fact been shown to have such properties^79,80^, the surprise signal account for the role of TBA in action control does not clearly detail the basis of this comparison, i.e., to which representation incoming information/feedback is compared to. Considering BRAC and its reliance on event-files that have been conceptualized as episodic memory traces^43^, the comparison processes leading to the surprise signal may well depend on the event-file information that is retrieved at a particular time point. In this respect, BRAC does not contradict the surprise signal account of TBA in action control. Rather, it further specifies central aspects leading to the TBA surprise signal in an already validated theoretical framework. According to the BRAC logic, analyses of neurophysiological data in action control paradigms almost exclusively focus on the analysis of processes following the presentation of the target (probe) stimulus. The typical so-called ‘action effects’ (i.e., the consequences of an executed action) emerging in action control tasks are, according to BRAC, always a composite of binding proper and retrieval. From the perspective of BRAC, the role of TBA in action control can therefore be due to its role in binding and retrieval processes of event-files. However, since BRAC assumes that binding and retrieval processes independently influence event-file dynamics and thus the outcomes of an action, the important question is whether TBA is more important for binding or retrieval processes or whether TBA equally contributes to these processes? At present, this question cannot be answered, but it appears as if the latter scenario is likely. For instance, the surprise signal may be present particularly in prime-probe sequences, where the retrieved event-file doesn’t match the to-built event-file at the probe. That is, in cases of partial matches between prime and probe, stimulus and response features are not in keeping with the retrieved organization in the prime event-file and therefore have to be reorganized. Aside a prominent role in action control, TBA also plays an essential role in working memory processes^81–83^. Action control processes and working memory processes are inter-dependent^84,85^, which is corroborated by evidence from functional imaging^85–87^ and electrophysiological/behavioral studies^88–91^. Available evidence from working memory research suggests that TBA is particularly relevant during the sequential coding of working memory items^82^. This sequential (episodic) structure of working memory encoding has strong similarities with event-file coding processes^15,92,93^ central for BRAC^6^ encompassing a memory trace specifying stimulus-response associations^43^. From that perspective, and considering BRAC also stressing the role of sequential prime-probe processes in commonly used experiments to measure action control, the role of TBA or surprise signals during action control may root in binding and retrieval processes and thus in the dynamic management of event-files reflecting episodic memory traces. This also well-reflects evidence that TBA may reflect “internally-driven” cognitive functions^94,95^. However, as regards the sequential ordering of information in working memory, not only TBA, but also gamma band activity is implicated^82^. Interdependencies of theta and gamma band activity are a hallmark of hippocampal circuits and fronto–hippocampal networks^96,97^. Similar evidence comes from the field of episodic memory processing^98^, where studies revealed a role of hippocampal theta-gamma synchronization for the encoding and retrieval of episodic memories. Of note, an intricate relationship of theta/gamma synchronization and desynchronization processes between cortical and subcortical structures is also relevant to consider for episodic memory processing^98^. Likely, gamma band activity is coordinated by an underlying theta rhythm in memory processes^82,96,97^, which puts TBA in a central position for mechanisms conceptualized in BRAC to understand goal-directed behavior.

When touching the role of gamma band activity (GBA) in relation to TBA, the question arises whether gamma band activity can be conceptualized in the BRAC framework. The answer is: partly. “Binding”, a core concept for BRAC and event-files, also plays a central role in contemporary conceptions of GBA^99–101^ due to its role in information transfer across the cortex^102,103^. Especially the phase of GBA is thought to enable the synchronization of neural firing within a cortical region^60,63^. Crucially, and due to biophysical peculiarities of high-frequency oscillations, such binding processes are confined to circumscribed cortical regions and play an essential role in sensory object representation and processing^104^. Because event-files can be conceived as a network of feature representations^43^ spanning multiple cortical regions^69,105–107^, GBA is unlikely to play a role in all aspects of event-file binding. Yet, it can well play a role in constituting the representations of sensory information within an event-file (note: the event-file contains sensory feature representations that are bound to motor feature representations). Indeed, evidence suggest a role of GBA in event-file bindings^108^.

### Alpha band activity in the light of BRAC

The above discussion about the possible functional conceptualization of TBA using the BRAC framework already touched the fundamental concept that event-files, as one building block of BRAC, reflect episodic memory traces^43^. The event-files, and especially binding and retrieval processes therein, putatively mediated through TBA, have to be managed in a dynamic fashion to accomplish successful action control on the basis of perception-action integration processes. However, when considering the dynamic management of episodic memory traces (such as event-files) alpha band activity (ABA) comes into play. Likely, the interplay of TBA and ABA is essential for the dynamic management of event-files as an emerging mechanistic principles of goal-directed action control. Probably the most prominent conceptualization of ABA function is the ‘inhibition timing hypothesis’^109,110^ assuming a special role of ABA, because ABA is the only frequency band, in which event-related synchronization (ERS) and desynchronization (ERD) processes (i.e., increase or decrease in power) occur in response to stimulus information or task demands. According to the inhibition timing hypothesis, rhythmic fluctuations of alpha oscillations gives rise to increases and decreases of inhibition that may equal a selective activation of inhibition of certain aspects during information processing^109^. It is the timing and direction of a change in inhibition – described by phase – that is functionally related to the timing of the inhibitory control processes. Alpha ERS reflects the inhibition of task-irrelevant information^109^ regardless of the sensory modality. Due to the multi-modal nature of event-files^43^ this is relevant, because not all bound features in an event-file are always helpful, when an event-file is activated and retrieved during action execution. In fact, irrelevant features can impede goal-directed behavior. One of the most robust and well-replicated findings is that the strength of bindings between features in an event-file is indexed by their behavioral costs or benefits these event-file bindings unfold whenever an event-file is activated and retrieved^15,73,74,111,112^. Crucially, due to the network-like structure of event-files^112^, the activation of an event-file can occur rather automatically whenever a feature is encountered that has previously been integrated into an event-file. Thus, a critical aspect is how to gate or control the activation of event-files during their dynamic management. According to BRAC, this is achieved through top-down or bottom-up attentional functions. Findings suggests that especially alpha ERD^109^ may reflect attentional control, however, also TBA has been brought into connection with top-down attentional control^113,114^. In this respect, the alpha mechanism is directly linked to mechanisms supposed to modulate the dynamic management of event-file binding and retrieval processes in the BRAC framework^6^. Importantly, BRAC and its event-file concept can also well capture what is being controlled by ABA’s inhibitory gating properties. It has been argued that the importance of ABA is not restricted to a specific “cognitive domain” such as perception, attention, working memory, and long-term memory^109^, so that ABA can control access to a “knowledge system” containing integrated information. Crucially, the event-file concept is neither restricted to working memory or long-term memory processes. It reflects a structure containing episodic information^15^ modulated by attentional processes^6,115,116^. There is a prominent role of ABA synchronization and desynchronization processes in episodic memory retrieval^98^. Several lines of evidence have substantiated that alpha/beta desynchronization reflect information processing during the perception of an event and also predict how well this information can be retrieved later on^98^ when alpha/beta desynchronization is also evident. ABA may therefore be central to coordinate the binding and retrieval dynamics of event-files as conceptualized in BRAC^6^ to adapt behavior, which also well reflects evidence that ABA possibly reflect “externally-driven” cognitive functions^94^. To ensure a coordinating role in the dynamics conceptualized in BRAC, ABA has to closely interact with and modulate TBA that, as outlined above, is relevant for binding and retrieval processes.

Indeed, much evidence reviewed in detail elsewhere^109,117,118^ corroborates that ABA plays a key role in the structure of oscillatory dynamics in the brain. This is likely due to physical properties (i.e., harmonic relations) between the alpha, theta and beta frequency band. Especially harmonic frequencies allow a close “communication” between frequency bands^109^ through cross-frequency coupling^62,119^. Of note, the commonly dissociated frequency bands (especially theta and beta) are organized and centered around the alpha frequency band Given the central frequency of ABA at 10 Hz, the central frequency of TBA is at the harmonic frequency (f) f_α_/2 = 5 Hz. For the beta frequency band, the central frequency is at the harmonic f_α_*2 = 20 Hz. These considerations suggest that cross-frequency couplings between TBA, ABA and also beta band activity (BBA) and thus the interactions of different cognitive subprocesses reflected by the individual frequency bands are essential for analyses. This would also be very much in line with the conceptual impetus of BRAC, according to which the individual processes are separable, but only the interaction of these processes can really explain action control in a sufficient way. While there are clear reasons for cross-frequency coupling analyses from a neurobiological and biophysical perspective^120^, there is as yet little cognitive science justification for this to better understand how action control unfolds. With respect to ABA, this is likely to change, though, considering the BRAC concept and a stronger connection between cognitive theory and neuroscience aspects coming in range through the choice of data analysis methods^5^.

### Beta band activity in the light of BRAC

When discussing the role of ABA and its interactions with other frequencies, the role of BBA (~14–25 Hz) already became apparent. BBA is typically assumed to be involved in sensorimotor processing^121,122^. The amplitude of beta oscillations across sensorimotor areas decreases just prior to and during movement execution. Conversely, an increase of beta amplitude above baseline levels is observed following movement execution, which has been referred to as post-movement event-related synchronization [ERS]^123^. Beta oscillations have the tendency to fluctuate during movement. Generally speaking, movements decrease beta activity^124^, while successful movement cancelation typically increases beta activity^125–128^. Movements do not even have to be executed for BBA to decrease. Even planned movements have an impact on beta power. This makes BBA fit exceptionally well to the action control literature and the BRAC framework. The processes described by BRAC are focused on the ‘planning’ aspect of actions and not the monitoring of movement execution. Of note, the concrete effector (e.g., feet vs. hands) or the movement type do not seem to affect BBA^124^, which again is in keeping with its goal-related action representation as emphasized by BRAC and TEC.

However, the exact functional role of BBA is still under debate^124,125,129^. One interpretation of beta ERS corresponds to cortical removal of excitation or idling^130^. Alternatively, beta ERS has been suggested to reflect an active inhibition of the motor cortex by somatosensory feedback^131^. Post-movement ERS has been interpreted more specifically as an indicator of movement outcome processing^122^. Supporting evidence stems from findings showing post-movement ERS to be modulated by passive movements^131,132^ and by kinematic errors^133^. More recently, Tan et al.^134^ reported that the level of post-movement ERS over the sensorimotor cortex serves as an index of confidence in the prediction of a motor outcome. Finally, and probably most prominent, Engel and Fries^135^ proposed that beta synchronization signals the tendency of the motor system to maintain the sensorimotor set or ‘status quo’. In this sense, beta ERS might be related to temporal representations of individual actions, of the sort discussed in the action control literature. Against the background of BRAC, post movement beta ERS is related to short-term stimulus–response bindings. Pastötter et al.^136^ suggested that post movement beta ERS is related to individual differences in the short-term storage and decay or disintegration of event-files. They found that beta ERS and distractor-based binding effects correlate such that the stronger the BBA the longer event files could impact upon subsequent actions. Indeed, the idea that beta ERS is related to such short-term memory function is in line with previous studies linking BBA to short-term attention and working memory^137,138^. In this regard, a recent conception of BBA^129^ suggests a more fine-grained interpretation of the status quo idea. In fact, instead of maintaining a representation, this concept assumes content-specific BBA that changes from active to latent to re-activated states. The transition from latent to re-activated cortical representations can be driven endogenously^129^ but might also be induced by feature repetitions. Within BRAC terms, repeating a feature from one episode to another might actually reactivate content-specific BBA (and might also show interactions with TBA). More generally, the sequential structure of action control tasks as adapted by BRAC clearly fits in the short-term storage and decay aspects of BBA. After responding to a trial, post movement BBA may reflect the maintenance (or transition from active to latent memory) of the trial’s event file and the strength of BBA may indicate the time window, in which this event-file can potentially be re-activated. This interpretation of BBA specifies the ‘status quo’ interpretation of Engel and Fries^135^ and is in line with more recent concepts of BBA^129^. Dynamic management of event-files is not only required in experiments analyzing binding and retrieval processes but occurs in every task or situation where humans rapidly act several times in succession. BBA of different event-files might thus reflect the traces of actions that must be handled by the cognitive system when feature-repetition suggests retrieval of previous instances. This holds true for observations of BBA in multi-item working memory studies that found bursts in BBA when individual working memory items were repeated^139^. It also relates to findings in decision making tasks, where it has been argued that BBA reflects the dynamic updating of information and the mapping of this information to a motor response.

#### A synthesis of oscillatory activity during action control using the BRAC concept

**Fig. 2:**
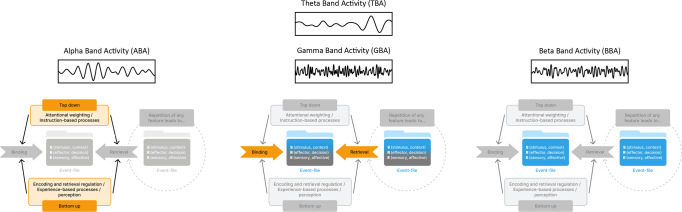
Oscillatory activity systematized using BRAC. Oscillatory activity in the theta, alpha, beta and gamma frequency band mapped on the mechanistic structure of the BRAC framework. Alpha band activity is likely to be central for top-down (i.e., attentional weighting/instruction-based processes) and bottom-up (i.e., experience-based processes/perception) modulatory effects on binding and retrieval processes. Theta band activity is thus directly modulated by alpha band activity, because theta band activity is supposed to reflect binding and retrieval processes impinging on event-files Gamma band activity is also part of this dynamics, especially as far as the encoding/retrieval of locally bound stimulus features underlying a coherent perception are concerned. Beta band activity is thought to be essential for constituting the event-file structure and is thus subject to theta/gamma band activity effects underlying binding and retrieval processes.

BRAC offers the possibility to integrate functional interpretations of oscillatory activity and their inter-relation (see also Fig. 2):

Taking the BRAC perspective, TBA likely reflects the binding of sensorimotor features into event-files and information integration during retrieval. Especially when stimulus information is translated into motor codes, retrieved S-R couplings may facilitate or hamper feature binding. Thus, TBA reflects the dynamic integration of retrieved and to-be-build event-files with TBA particularly increasing when demands on cognitive control are high, i.e., event-file reconfigurations is required. This BRAC-interpretation can readily be reconciled with biophysical principles of TBA facilitating the integration of information between distant functional neuroanatomical regions^59^ and currently assumed computational principles of TBA likely reflecting a “surprise signal” that initiates adaptive processes^77^. Crucially, the fact that event-files managed by processes defined in BRAC reflect episodic memory traces also connects research on the role of TBA in action control, predominantly implicating cortico-striatal loops, with research on the functional significance of TBA in memory-related processes in functional neuroanatomical structures including the hippocampus and related structures.

Crucially, also GBA, currently considered to play only a minor role in action control, becomes an integral part through its role in “binding”^99–101^ as a core concept of event-files and BRAC-dynamics. Binding and retrieval of integrated sensori-motor representations (event-files) are independent factors determining event-file management^6^. It appears that whereas GBA-TBA interaction are particularly important in the binding aspects of event-file dynamics and less so in the retrieval part of the event-file management dynamics, GBA likely plays a very specific role. Thus, GBA may be of particular importance for the representation of sensory information constituting one part of the event-file information, in line with its essential role in sensory object representation and processing^104^. GBA is likely to be modulated by TBA during event-file binding processes in action control. This is substantiated by abundant evidence that GBA is coordinated by an underlying theta rhythm in (episodic) memory processes^82,96,97^. Considering the evidence that GBA plays a central role, in how distinct features constituting a (e.g., visual) stimulus are bound into a coherent percept in sensory cortices^102–104^, it is plausible that GBA is involved in the sensory aspects of an event-file and probably central for the coherent representation of sensory information within an event-file. Crucially, since in most widely used experimental approaches examining action control analysis of neurophysiological processes are focused on the “probe” information with little emphasis on the role of perceptual processes as modulators of action control during event-file binding, it is perhaps not surprising that GBA has been neglected in scientific discussions about the neural basis of action control. The paucity of findings on GBA in action control is likely reflecting a bias in research on action control, which is, at least partly, attributable to the theoretical concepts currently motivating research in action control. The more holistic concept offered by BRAC is likely to change this with the effect that research on GBA will become connected more closely to research in other frequency bands already playing a central role in the cognitive neuroscience of action control.

Whereas dynamics of TBA and GBA during action control may have their relevance in binding and retrieval processes of integrated sensorimotor representations (event-files), the role of ABA is likely related to the modulation of binding and retrieval dynamics and may interfere with TBA and GBA dynamics. ABA probably predominantly reflects the top-down and bottom-up attentional modulation of binding and retrieval processes within the BRAC framework, which is in line with the well-established conception of inhibitory gating processes exerted by ABA^109,110^. The latter appears to be relevant for the inhibition of irrelevant features potentially impeding goal-directed behavior and may thus crucially coordinate binding and retrieval dynamics of event-files. Taking this perspective, ABA is likely interacting with TBA/GBA associated mechanisms during event-file binding and retrieval processes given that BRAC assumes that top-down and bottom-up attentional modulation can independently modulate binding and retrieval mechanisms^6^. Taken together, TBA, ABA and GBA are likely not directly relevant for the structure of an event-file—that is the processes constituting the maintenance/stability of features in an event-file. These aspects may be a function of BBA. Because, as pointed out, event-files are traces of episodic memory, it is conceivable that event-files are established through the interplay of TBA, ABA and GBA reflecting the relation between binding and retrieval. Especially during retrieval, a previously established but inactive event-file might be re-activated, that is, according to a recent BBA framework^129^ the transition from latent to re-activated cortical representations, as reflected in BBA, might be induced by feature repetitions. Within BRAC terms, repeating a feature from one episode to another might thus actually reactivate content-specific BBA (and the respective event-file content) that must then be integrated with the processes and brain oscillations building the current event-file (suggesting an interplay between BBA and TBA). Thus, BBA, although less clearly defined as TBA and ABA, probably reflects the integration duration of event-files pointing at a role in the handling of action traces. BBA might reflect the re-activation of latent bindings between represented features, which fits exactly to the BRAC logic of keeping an event-file accessible for later retrieval. Event-files, however, decay over time and might not be retrievable when memory traces become too spurious.

The discussion thus far was centered around the role of oscillatory activity in different frequency bands and their functional relevance in terms of the processes specified in BRAC, without strongly touching the aspect about the functional neuroanatomical implementation. Nevertheless, the functional neuroanatomical level is also implied because theta and gamma band activity strongly refer to these activities in specific functional neuroanatomical structures (see section on theta/gamma band activity). Previous studies on event file coding as conceptualized in TEC, have consistently revealed that event-file dynamics reflect processing in a distributed cortical and subcortical network encompassing inferior and superior parietal areas, supplementary motor areas, the dorsolateral prefrontal cortex, and the hippocampus^69–76^. For alpha and beta band activity, however, the likely relevant functional neuroanatomical implementation is more contentious, even though some studies have provided evidence that inferior and superior parietal structures are involved in alpha band activity during event file coding^68,140^. From the evidence outlined above it appears that a fronto-parietal network is involved in the dynamics central to assumptions of BRAC. However, particularly the involvement of prefrontal regions cannot be seen in isolation from subcortical (basal ganglia and cerebellar) structures, since these are closely connected with prefrontal regions^141^ and involved in cognitive processes^142,143^ that BRAC seeks to conceptualize. Of note, these structures are also involved in the integration of perception and action^144^ and thus basic ideomotor principles^7^ reflecting the conceptual origin of BRAC. The specific contribution of these structures in relation to oscillations in different frequencies and their possible functional relevance within the BRAC framework should be addressed in the future (see Box 2).

#### Further conceptual implications for neurophysiological research methodology

While the BRAC framework conceptualizes the interplay of oscillatory activity in different frequencies in terms in terms of their cognitive function, it is important to mention that another important aspect of brain physiological activity may also able to do so – the so-called global signal (GS)^145^. GS has a specific physiological basis which mediates the level of arousal and coordinates the cortical regions’ and networks’ activities, thereby organizing different forms of cognition^145^. It has been argued that GS is driven by the infra-slow fluctuations upon which the activity of faster frequencies is organized through phase-amplitude coupling. As reviewed elsewhere^145^, especially delta/theta band activity and gamma band activity contribute to GS. In contrast, the alpha/beta range is not related to GS^145^. From that perspective and given the above line of arguments (see also Fig. 2), GS is relevant for TBA/GBA-associated binding and retrieval processes impinging on event-files. Yet, GS is probably less relevant to processes possibly central for ABA-related top-down and bottom-up modulatory effects on binding and retrieval processes, or BBA-associated processes essential for constituting the event-file structure.

An important implication of BRAC – especially when it comes to the analysis and interpretation of neurophysiological data – is the sequential structure of events with the consequence that an action can serve as a cue that another affordance requiring another action is likely to come up. BRAC’s central aspect that the “immediate past” shapes current behavior^6^ must be extrapolated to the structure of experiments and their “inter-trial / trial” structure. Possibly the mere sequential structure of trials in experiments (even if trials are presented random or pseudo-randomly) is sufficient to induce re-iterant states of neural activity, which has major consequences:

First, cognitive processes underlying action control are likely pre-determined by the system’s neural state in periods commonly considered unimportant for the understanding of cognitive functions (i.e., the inter-trial interval). Therefore, knowledge accumulated so far about the neurophysiological processes underlying action control via the traditional data analysis focus in experiments is systematically biased and that accumulated knowledge based on these procedures must be re-evaluated. Indeed, there is ample evidence that neurophysiological activity in theta^146^, alpha^140,147^, beta^148^ and also delta frequency bands^149^. Relatedly, second, it is common practice to use the neurophysiological signal (e.g., in the EEG) during inter-trial intervals as “reference” for the quantification of neurophysiological activity during specific cognitive processes for which the experiment was designed. Crucially, the rationale behind this baseline correction^150–153^ only refers to the power of neurophysiological activity, but does not consider the information being coded in that interval. Yet, it is the information being coded and not the power of a neurophysiological signal that is of relevance to the understanding for the cognitive functions of interest to an experiment. If the inter-trial interval contains information useful to forecast the spatio-temporal pattern of neurophysiological activity during cognitive processes of interest (i.e., within a trial), the evaluation of processes become circular/self-referential when being related to the inter-trial interval.

Box 2 Outstanding questions Is alpha band activity central for top-down (i.e., attentional weighting/instruction-based processes) and bottom-up (i.e., experience-based processes/perception) modulatory effects on binding and retrieval processes?

Is theta band activity directly modulated by alpha band activity, because theta band activity is supposed to reflect binding and retrieval processes impinging on event-files?

Is gamma band activity also part of theta dynamics, especially as far as the encoding/retrieval of locally bound stimulus features underlying a coherent perception are concerned?

Is beta band activity essential for constituting the event-file structure and is thus subject to theta/gamma band activity effects underlying binding and retrieval processes?

Is there a hierarchy of cognitive subprocesses and their associated oscillatory dynamics?

What is the precise functional neuroanatomical architecture associated with BRAC-related oscillatory activity?

## Outlook

Taken together, BRAC has the potential to systematize and re-structure the evidence and results of the rich literature on brain oscillations investigated in experimental tasks that focus on actions. It assigns clearly defined roles to TBA, ABA, BBA, and GBA across paradigms and thus offers a holistic view on the functional relevance of these brain oscillations and – importantly—their interrelations to better understand goal-directed actions and underlying subprocesses. The comprehensive and inclusive approach opens up paths in different directions in different fields of neuroscience.

### Reporting summary

Further information on research design is available in the Nature Portfolio Reporting Summary linked to this article.

## Supplementary information

Reporting Summary
